# Development and Initial Evaluation of a Digital Phenotype Collection System for Adolescents: Proof-of-Concept Study

**DOI:** 10.2196/59623

**Published:** 2024-10-24

**Authors:** Minseo Cho, Doeun Park, Myounglee Choo, Jinwoo Kim, Doug Hyun Han

**Affiliations:** 1 Human Computer Interaction Lab Department of Cognitive Science Yonsei University Seoul Republic of Korea; 2 Human Computer Interaction Lab School of Business Yonsei University Seoul Republic of Korea; 3 HAII Corp Seoul Republic of Korea; 4 College of Medicine Chung-Ang University Seoul Republic of Korea

**Keywords:** adolescents, adolescent mental health, smartphone apps, self-monitoring, qualitative research, phenotypes, proof of concept, digital phenotyping, phenotype data, ecological momentary assessment

## Abstract

**Background:**

The growing concern on adolescent mental health calls for proactive early detection and intervention strategies. There is a recognition of the link between digital phenotypes and mental health, drawing attention to their potential use. However, the process of collecting digital phenotype data presents challenges despite its promising prospects.

**Objective:**

This study aims to develop and validate system concepts for collecting adolescent digital phenotypes that effectively manage inherent challenges in the process.

**Methods:**

In a formative investigation (N=34), we observed adolescent self-recording behaviors and conducted interviews to develop design goals. These goals were then translated into system concepts, which included planners resembling interfaces, simplified data input with tags, visual reports on behaviors and moods, and supportive ecological momentary assessment (EMA) prompts. A proof-of-concept study was conducted over 2 weeks (n=16), using tools that simulated the concepts to record daily activities and complete EMA surveys. The effectiveness of the system was evaluated through semistructured interviews, supplemented by an analysis of the frequency of records and responses.

**Results:**

The interview findings revealed overall satisfaction with the system concepts, emphasizing strong support for self-recording. Participants consistently maintained daily records throughout the study period, with no missing data. They particularly valued the recording procedures that aligned well with their self-recording goal of time management, facilitated by the interface design and simplified recording procedures. Visualizations during recording and subsequent report viewing further enhanced engagement by identifying missing data and encouraging deeper self-reflection. The average EMA compliance reached 72%, attributed to a design that faithfully reflected adolescents’ lives, with surveys scheduled at convenient times and supportive messages tailored to their daily routines. The high compliance rates observed and positive feedback from participants underscore the potential of our approach in addressing the challenges of collecting digital phenotypes among adolescents.

**Conclusions:**

Integrating observations of adolescents’ recording behavior into the design process proved to be beneficial for developing an effective and highly compliant digital phenotype collection system.

## Introduction

### Background

Adolescent mood disorders have been on the rise in recent years, particularly in the aftermath of the COVID-19 pandemic, which has introduced significant disruptions to their lives [1-3]. These disruptions include changes in routine, social isolation, academic stressors, and heightened uncertainty about the future [4-6]. Combined with the natural challenges of adolescence, such as physiological and emotional changes, these factors contribute to an increased susceptibility to mood disorders [7,8].

The long-term implications of untreated mood disorders during adolescence are significant. Research indicates that untreated depression and anxiety during this period can have lasting effects on mental health and well-being into adulthood [9-12]. This includes difficulties in academic and social functioning, heightened risk of substance abuse, and an increased likelihood of developing chronic mental health conditions [13-15]. Early detection and intervention are vital to address these issues and minimize their impact on long-term outcomes [16-18].

However, identifying mood disorder symptoms in adolescents can be challenging due to differences in manifestation and the tendency to attribute them to typical adolescent behavior or mood swings [19-21]. Moreover, adolescents may be reluctant to seek help from traditional clinical services due to stigma, confidentiality concerns, or a lack of awareness about available resources [22-24]. This underscores the need for innovative and less intrusive methods of detection and intervention [25-27].

In recent years, there has been growing interest in using digital technologies and data collection methods to monitor and evaluate the mental health of adolescents [28]. Among these approaches, digital phenotype has emerged as a promising strategy [29,30]. This method involves gathering digital data, such as smartphone use patterns and social media activity, to infer the mental health status of individuals [31-33]. While digital phenotype holds promise for early detection and intervention in adolescent mental health, there are challenges associated with data collection that need to be addressed.

### Challenges in Collecting Digital Phenotypes

Passive digital phenotypes, which encompass various aspects such as location, activity, sleep, and smartphone use, have shown meaningful correlations with mental health states, facilitating the identification of risk levels and timely interventions [34-37]. However, collecting passive digital phenotypes presents several challenges. One significant issue is the potential for noise due to sensor dysfunction or disconnection, which can introduce inaccuracies in the collected data [38]. Moreover, passive digital phenotype collection systems often struggle to maintain consistent data collection as they rely on user interaction with the technology [39,40]. Additionally, labeling collected data presents difficulties that may compromise the accuracy of the analysis with the lack of contextual information [40,41].

To address these limitations, active digital phenotypes offer a complementary approach. In active digital phenotyping, individuals actively engage in self-reporting their mental health, offering a more direct and subjective input regarding their state [42-45]. However, collecting active digital phenotypes also comes with its own set of hurdles. One major challenge is the burden placed on subjects, leading to low compliance rates and incomplete datasets [46]. Additionally, active digital phenotype data collection often relies on subjective self-reporting, which can introduce biases that can affect the data validity [47]. Furthermore, issues with timing and scheduling can complicate the process, particularly in methods such as ecological momentary assessment (EMA), where data must be collected in real-time throughout the day [40].

### Study Objectives

Examining actual adolescents’ recording behavior can be helpful in refining the digital phenotype data collection process. By observing how adolescents track their daily mood or lifestyle behaviors, researchers can gain valuable insights into their preferences, habits, and challenges in engaging with digital technology for self-reporting purposes. This firsthand understanding will allow the tailoring of data collection strategies to better align with adolescents’ natural behaviors, fostering sustained engagement and accurate datasets. Ultimately, this approach is expected to enhance the relevance and usability of digital phenotypes in assessing mood disorders and other mental health issues among adolescents, contributing to more targeted and impactful interventions.

## Methods

### Procedure

The aim of our formative investigation was to understand what adolescents typically record and how they do it. Our study involved 34 students from middle and high schools in South Korea, comprising 23 (68%) female and 11 (32%) male adolescents aged 13 to 18 years. Participants were tasked with self-recording their lives for 3 days using mailed sheets. No specific instructions were given to genuinely explore each participant’s personal style of self-tracking. The self-recordings had to cover 2 weekdays and 1 day on the weekend to capture variations in adolescents’ daily routines due to school schedules.

Subsequently, in semistructured interviews, participants discussed their typical self-recording process, including motivations, strategies, benefits, and the possible need for assistance. They also engaged in reflective discussions regarding their experiences with the self-recording activity. These reflections encompassed considerations of their emotional states, levels of satisfaction, and overall well-being, allowing for nuanced assessments of their self-recording journey. This approach allowed us to gain insights into adolescents’ self-recording habits and preferences, shedding light on their natural inclinations and challenges in engaging in self-recording activities.

### Ethical Considerations

Ethical approval for the study was obtained from the Yonsei University Institutional Review Board (7001988-202304-HR-1711-06). After the introduction to the study, participants provided informed consent through phone calls or text messages. Participation was voluntary, and individuals could choose to opt out at any time. To protect privacy, all collected data were deidentified, with participants assigned identification numbers (eg, P1) based on their registration order. Upon completing the 3-day recording and interview process, participants were compensated with the cash equivalent of KR ₩100,000 (US $74.6).

### Study Results and Design Goals

Drawing on the thematic analysis of the interview and feedback from adolescents, we identified key ideas to support daily recording effectively. We mapped each challenge with the corresponding design goal as follows (Table 1).

**Table 1:** Design goals and relevant system concepts.

Design goals	System concepts
Align with adolescents’ self-recording goal	Overall purpose of time management Interface resembling a digital planner Records organized in a timetable format
Reduce user burden with simplified recording	Click-based recording process Tags reflecting adolescent’s life activities Short EMA^a^ survey questionnaire
Support self-awareness with data interpretation	Visual reports with feedback Deeper interpretation on recorded data Illustration on behavior-mood interaction
Provide social support and a sense of relatedness	EMA prompts scheduled to adolescent’s daily routine and opportune moments. Supportive phrases in EMA prompts

^a^EMA: ecological momentary assessment.

#### Time Management Support

Adolescents were frequently engaged in self-recording as a means to manage their time, with the goal of improving their academic performance and achieving a balanced lifestyle. Their recordings often encompassed various aspects of daily life, including study habits, leisure activities, social interactions, and sleep patterns, reflecting their intertwined priorities of academic success and well-being maintenance. Therefore, it is imperative for our system to center its efforts on time management, in line with adolescents’ motivation to track their behavior and mood. Additionally, considering the diverse nature of adolescents’ routines, our system should encompass a broader range of life-related activities.

#### Simplified Recording Process

The importance of simplified input processes was underscored by adolescents’ reluctance to manually record their daily activities. They expressed a preference for self-recording tools with shortcut functions like duplication, click-based recording, and automatic tracking. Interviewees also favored interfaces familiar to them to minimize learning burdens. Therefore, our design goal aims to integrate shortcuts for recording and use interfaces resembling digital planner apps generally used by adolescents. This strategy aims to reduce the effort needed for users to adopt and interact with the digital self-recording services, ultimately enhancing engagement and usability.

#### Visualizations and Feedback

Adolescents’ inclination toward visualization and feedback noted their aspiration for insightful reflections. They preferred concurrent recording and visualization to promptly confirm raw data. For deeper analysis, they sought visualization and feedback to aid in interpreting raw recorded data and gain further insight into themselves. These useful benefits played a pivotal role in maintaining adolescents’ engagement in self-tracking. In line with these insights, our design goal prioritizes delivering visual reports unveiling adolescents’ behaviors and moods, complemented by personalized feedback to nurture their self-recording journey and foster positive behavioral changes.

#### Social Support and Interaction

Adolescents were inclined toward engaging in self-recording activities with social elements. Social dynamics, including peer norms and support, were heavily influencing their self-recording behaviors. Notably, some interviewees shared their involvement in group chats aimed at mutual motivation for consistent tracking and feedback provision. Drawing from these insights, we have formulated a core design objective centered on fostering a sense of connection. Our aim is to serve as a supportive companion in adolescents' pursuit of self-recording, primarily focusing on time management for enhancing academic performance and maintaining a balanced lifestyle.

### System Design

Drawing on the thematic analysis of the interview and feedback from adolescents, we identified key ideas to support daily recording effectively. We mapped each design goal with the corresponding system features as follows.

#### Planner Interface

In order to encourage voluntary engagement, our primary focus was on aligning with interfaces familiar to adolescents. This entailed examining designs of frequently mentioned tools from interviews and integrating them into the development of our system’s concept, ensuring resonance with the preferences of adolescents. A commonly adopted format involved a timetable divided into 24 hours, allowing adolescents to log activities in corresponding columns. The time unit ranged from minutes to hours, offering flexibility for users seeking either detailed records or a generalized overview of their behavior. To streamline data input and reduce user burden, our system defaulted to 30-minute intervals, requiring entry of life activities in 48 spaces.

#### Tag Shortcuts

Observing the adolescents’ tendency to use colors, stickers, or tags to categorize similar activities, we recognized the importance of categorizing functions for recording purposes. After reviewing the self-recording sheets provided by adolescents, we noted that life behaviors could be broadly categorized into 4 areas: sleep, exercise, leisure, and study. We then created subcategories by analyzing self-records collected previously and creating frequently reported themes (Table 2). For instance, study could be recorded as “self-study,” “academy class,” “web-based class,” and “afterschool class.” We incorporated these classifications as recording tags with color-coding applied, addressing the perceived inconvenience associated with typing when using a digital planner.

**Table 2:** Categorized tags for daily life recording.

Category	Tags for recording
Sleep	Sleeping and napping
Exercise	Walking, aerobic exercising, weight training, and others
Leisure	Socializing, reading, movie going, traveling, smartphone use, gaming, watching TV, and others
Study	Academy class, web-based class, afterschool class, and self-study,

#### Visual Reports

Adolescents welcomed the visualized data as effective motivators for engagement, particularly when using digital planners or schedulers. In response, our system was designed to provide statistics covering total amounts and averages, alongside evaluation of the records to personal goals and objective standards.

Our formative investigation revealed that adolescents record their behaviors to manage study time effectively and maintain a balanced lifestyle. However, emotional aspects were relatively neglected in their recordings. Despite this oversight, emotional challenges often arose and affected their self-recording objectives, leading to decreased motivation for studying and resorting to risky behaviors such as increased media consumption. Building upon these insights, we devised methods to encourage adolescents to recognize the value of tracking their emotional states to progress toward their goals. Specifically, we illustrated how mood relates to behaviors in visual reports.

#### Supportive EMA

Based on the interview insights, we developed an EMA survey tailored to adolescents. Considering that adolescents face restrictions on smartphone usage during school hours, we scheduled surveys to avoid these inaccessible times and increased their frequency in the early morning and afternoon, totaling 8 times a day. Furthermore, to ensure the ease of response, we incorporated a 5-point Likert scale for evaluating depression, anxiety, and stress levels.

When prompting responses, we tailored our messages to align with the daily routines of adolescents. Morning messages were supportive, aiming to set a positive tone for the day with phrases like “Have a great day at school.” Around 3 PM, we inquired about their school day, acknowledging their midday experiences. Before academy classes, messages aimed to uplift them with phrases like “Cheer up!” offering social support in line with the preferences of adolescents.

## Results

### Prototype

**Figure 1:**
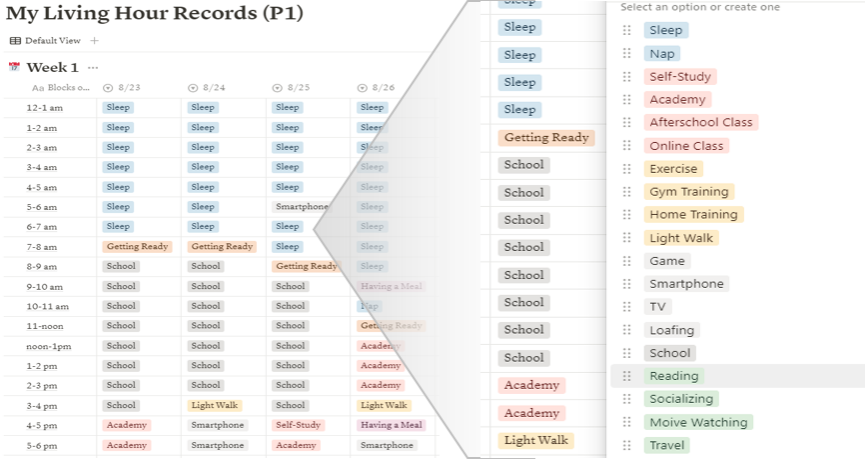
Simulation of a recording interface using the Notion website.

In our assessment of the active digital phenotype collection system concepts, we conducted simulations to evaluate its suitability within the everyday lives of adolescents. This proof-of-concept study involved researchers developing tools to replicate essential concepts. When simulating the recording interface, our priority was to design a user-friendly layout similar to digital schedulers commonly used by adolescents. We chose the Notion website, as it can feature a planner-like layout with dropdown options for daily self-recording. Through this simulation, we aimed to evaluate the usefulness of the interface and the adequacy of predetermined recording tags (Figure 1).

In exploring EMA design, we sought tools compatible with the participants’ smartphones to embed survey questionnaire links and send random notifications. This functionality was achieved using the reserved message feature of KakaoTalk (Kakao Corp), a web-based message service widely used in South Korea. Through this simulation, we aimed to assess the acceptability of the friendly prompt, scheduling frequency (8 times), and random timing for notifications. When simulating visualization and feedback, we provided manually created images summarizing recorded data, behavioral trends, and adherence to personal goals or lifestyle recommendations. Through this simulation, we aimed to determine whether visual reports enhance engagement in recording activities and assess the usefulness of the content included in the reports.

### Proof-of-Concept Study

During the proof-of-concept study, we aimed to evaluate the alignment of our system concepts with the ultimate goal of designing an effective active digital phenotype collection system and to gather feedback for improvement before advancing to the development of a working prototype. The study involved 16 middle and high school students from South Korea, 14 (88%) female and 2 (12%) male adolescents aged between 13 and 18 years. Participants engaged in daily recording of their daily life and mood for 2 weeks, followed by a 30-minute semistructured interview.

Each participant was provided with an individual Notion page for their daily life record. Additionally, they received daily mood recording prompts via KakaoTalk, enabling us to assess the timing, frequency, and expressions of the EMA surveys. There were no restrictions on participation for individuals with incomplete records, as this was considered indicative of potential usability issues. Participants who completed the recording period underwent semistructured interviews, where they shared their experiences with recording activities, discussed the system’s suitability for collecting active digital phenotype data, and provided insights for improvement.

### Study Results

The findings from our proof-of-concept study verified the effectiveness of the system concepts in collecting active digital phenotypes. Adolescents reported their motivation to voluntarily record their lifestyle behaviors and emotions, as evidenced by both qualitative feedback and quantitative data (Table 3). Moreover, the study provided insights into how the self-recording system influenced the management of lifestyle and emotions. as well as identified aspects necessary to tailor the system to meet the specific needs of adolescents.

**Table 3:** System features and quotes from participants.

System features		Sample quotes
**Planner interface**		
	1	“It [Interface] provided me a chance to see how I spent my time and reflect on myself every day.”
	2	“I could write in more detail about dividing an hour into 30 minutes.”
	3	“Seeing an activity continuously recorded in a vertical manner gave a sense of its duration.”
	4	“When I accessed [Notion page] to record and see the empty column, I wanted to fill it in.”
**Tag shortcuts**		
	5	“I found it convenient and clear to use tags since my daily life mostly fell into those categories.”
	6	“It was really easy to just click tags one by one with my phone. It took like 5 seconds.”
	7	“Seeing how my day was with those tags and various colors quite motivated me. It was fun.”
	8	“I don't think there was a mealtime or commuting on the tag, so I had to type it manually.”
**Visual reports**		
	9	“Since the report evaluates my day thoroughly, I felt like recording more to get feedback.”
	10	“After recording, it [report] helped me manage my mood, knowing why I was angry or sad.”
	11	“Based on the individual challenges, it would be good to suggest a personalized plans.”
	12	“Analyzing the sleep balance in the report helped me understand my sleep quality.”
**Supportive EMA^a^**		
	13	“It was comfortable to get a notification in the evening after the schedule was all over.”
	14	“I simply tried to remember what I was doing at that time and answered [EMA].”
	15	“I didn't feel overwhelmed with the frequency. The survey wasn't that lengthy either”
	16	“When I turned on my phone, I got an [EMA] alarm and it asked me how I felt. I really liked it.”

^a^EMA: ecological momentary assessment.

#### System Use

**Figure 2:**
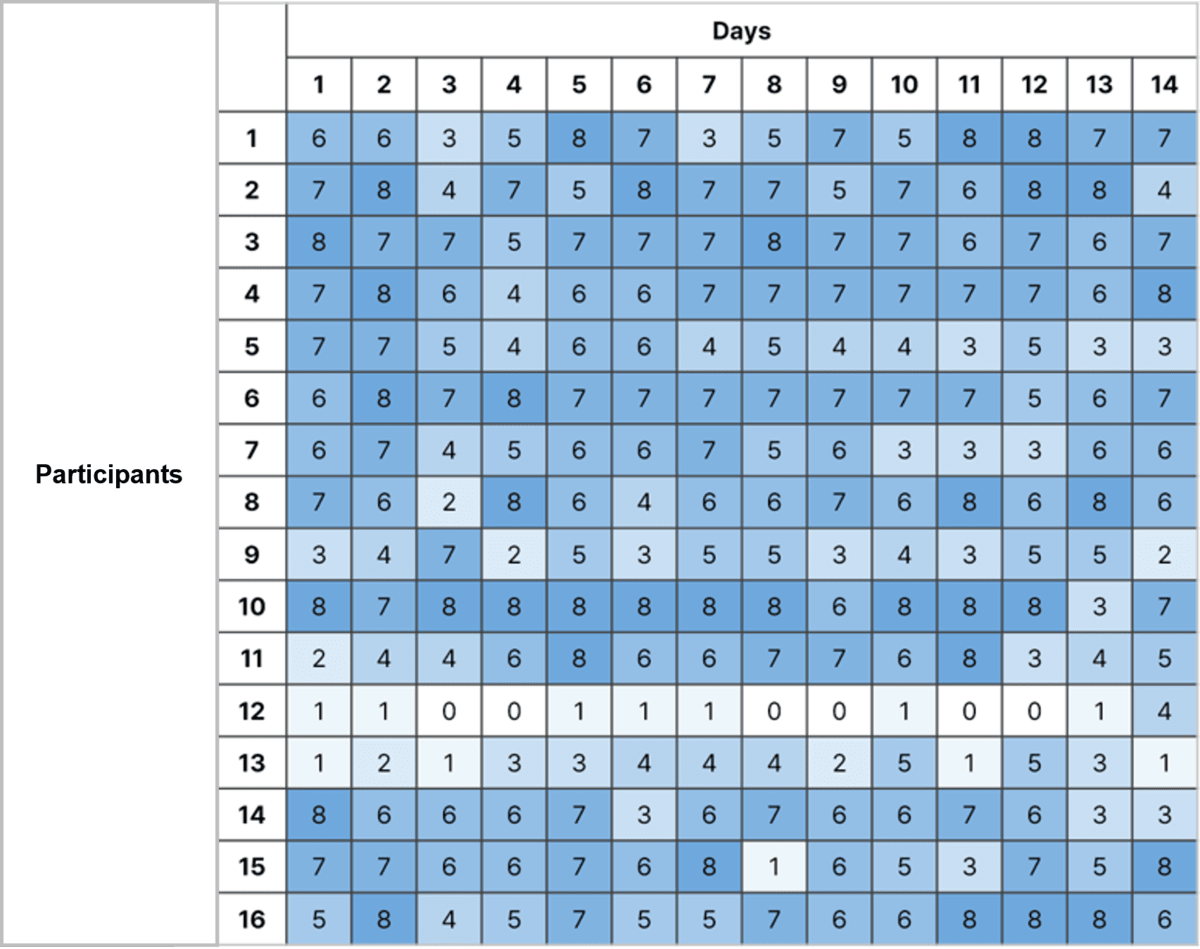
The number of responses to EMA by participants over a 2-week period. EMA: ecological momentary assessment.

In our study, 16 participants were instructed to complete 8 EMA surveys per day (Figure 2). One participant had a lower response rate of only 10%, providing 11 responses over 2 weeks. Considering this participant was an outlier, we excluded his data from further analysis. Upon further investigation during semistructured interviews, we discovered that the participant had misunderstood the study guidelines, believing he was only required to respond once per day. Excluding this outlier, the average number of EMA responses received over the 2 weeks was 80.6 (SD 4.2), with individual totals ranging from 39 to 103. When recalculated as a percentage, the overall response rate across participants was 72%, with individual rates ranging from 35% to 92%. Regarding compliance over the 14-day period, we observed a consistent average compliance rate of 72%, with a peak of 79% and a low of 62%. These findings suggest that our method of delivering EMA surveys was effective, maintaining stable compliance rates and sustained participant engagement over time.

In terms of self-recording, all participants recorded their daily activities for a 24-hour period over 2 weeks without any missed entries. Participants mentioned that the simplicity of the process, clicking tags to record, contributed to their overall high compliance. The recording was not burdensome and typically took less than a minute to complete. However, it is worth noting that not all entries were made promptly; rather, participants often completed them retrospectively. During follow-up interviews probing this finding, they admitted to forgetting or being unable to complete the daily record on certain days. When presented with a consecutive display of their records for 7 days, participants naturally identified missed records and completed them. The sight of the blanks seemed to trigger their impulse to fill them in, thereby contributing to prolonged engagement with the recording process.

#### Planner Interface

The validation of our planner-looking interface affirmed its user-friendliness among adolescents, seamlessly aligning with their habitual approach to recording their daily lives. Participants reported the easiness in navigating the Notion page interface, reflecting the advantage of familiarity and simplicity. This observation underscores the success of our design goal to create an interface that resembles commonly used digital planners and schedulers, thereby eliminating the need for the burden of learning new functions.

Moreover, as evidenced by the number of daily life records, the immediate display of daily life records in a planner format triggered adolescents to fill out missing records upon viewing them. While our planner interface initially divided the day into 24 columns, some participants suggested 30-minute intervals, to better accommodate their varied activities within 1 hour. They suggested making the time window selective, allowing users to customize the recording columns based on their preferences.

Our interface design not only boasted high usability but also demonstrated advantages in promoting self-reflection among adolescents. The vertical organization of records facilitated a visual representation of behavioral patterns over time, enabling users to gain insights into the continuity of their actions. Furthermore, presenting weekly records side by side offered a comprehensive overview of lifestyle habits, aiding in the identification of balanced activity distributions.

#### Tag Shortcuts

The structured categorization of recording tags into activities such as sleep, exercise, leisure, and study proved instrumental in simplifying the life-logging process. Feedback from participants highlighted the convenience of predefined options, which streamlined the recording process by simply requiring them to select the appropriate tag. Furthermore, these categorized tags helped them organize their daily routines by assessing whether each category’s distribution aligned with their ideal amount. Naming and labeling each hour by category also aided in planning for upcoming days.

While our tags adequately captured the daily routines of adolescents based on the self-records gathered during the formative study, participants noted the absence of tags for essential life activities such as “taking a shower,” “having a meal,” and “commuting.” Despite this, some participants expressed satisfaction with the provided tags but suggested the option to create manual ones. Additionally, the use of specific colors for each life area provided immediate insights into dominant behaviors, aiding in the assessment of activity distribution and patterns.

#### Visual Reports

The report played a significant role in enhancing adolescents’ self-awareness by integrating visual elements into their records. While self-recording alone allowed adolescents to capture aspects of their lives and emotions, the report took a step further by using visualizations like graphs and charts to render the recorded data more engaging and accessible. This heightened self-awareness prompted reflective practices, leading to the development of plans and actionable steps. However, the extent of behavior changes varied depending on individual factors such as motivation and self-regulation level. Consequently, some participants expressed a desire for reports to take on a more active role, such as collaborating on goal-setting and suggesting action plans.

Beyond the data directly available from the records, the report provided adolescents with a deeper understanding of themselves. Discovering previously unrecognized aspects of themselves proved beneficial, with observed positive changes acting as motivation for increased participation in self-recording. Within the mood report components, the researcher chose not only to display changes in depression, anxiety, and stress scores reported by adolescents but also to present recorded behaviors alongside these scores. This approach encouraged adolescents to consider contextual factors simultaneously when experiencing emotional issues, offering an opportunity for reflection on the causes of emotional problems and nurturing an understanding of the connection between behaviors and emotions.

#### Supportive EMA

In collecting mood records through the EMA approach, our goal was to validate the frequency and timing of survey notifications. Results indicated that receiving 8 notifications per day was well-received by participants, with high compliance observed. This supports our design goal to balance the data collection needs with participants' preferences and engagement levels.

Adolescents preferred receiving notifications in the evening when they had more available time to respond promptly. Moreover, they felt a sense of care from the system and valued the supportive prompts, especially enjoying the encouraging messages after school when they were feeling most fatigued. This suggests the potential interventional role of EMA prompts in assisting adolescents in managing their mood and building a rapport with the system. Furthermore, these findings lead to the importance of considering users’ daily routines in designing EMA, which can enhance overall user experience and participation.

Despite occasional delays in responding to surveys due to various contexts, adolescents demonstrated retrospective reflection on their emotions. This underscores the need for additional features that assist adolescents in reviewing their days for reliable retrospective responses on EMA surveys. When responding to past EMA surveys, participants primarily recalled the context, people they were with, or activities they were engaged in at the time the survey was initially provided. To enhance accuracy, it could be beneficial to first include questions oh these relevant features that help recall the context of that time.

## Discussion

### Principal Findings

Through a formative investigation, we derived system concepts aimed at effectively collecting active phenotypes. By exploring adolescents’ self-recording habits, tool preferences, and engagement factors, our system integrated a user-friendly recording process and accessible EMA features. The proof-of-concept study affirmed the efficacy of these concepts in collecting active phenotypes from adolescents. Participants favored the planner-type recording interface and found the predefined tags convenient. The immediate visualization of their records in a planner format prompted participants to fill in missing data, enhancing overall engagement. Additionally, participants valued the practical benefits of visual reports, which motivated them to provide more records for comprehensive visualization and feedback. The EMA design, with its adolescent-friendly scheduling and supportive prompts, received positive feedback.

Furthermore, we uncovered potential links between mental health monitoring and intervention. Self-recording could enhance adolescent self-awareness, facilitating the development of management plans and actionable steps. Visual reports on recorded life and mood held promise in aiding this process, and discussions on the use of report contents underscore the importance of tailoring content to reflect the preferences of adolescents for each life domain. Additionally, EMA prompts could provide momentary support tailored to adolescents' routines, potentially alleviating mood issues.

The interventional possibilities of the EMA prompts were in phrases reflecting the daily lives and behaviors of adolescents. Unlike generic messages, these prompts conveyed a sense of personal interest in the adolescents which could enhance feelings of companionship and support. To further improve this potential, future EMA prompts could be dynamically adjusted based on the recorded daily activities of adolescents. For example, when the recorded sleep time is below the recommended amount, the prompt may ask adolescents if they are tired. This customization can be made even more precise by using passive digital phenotypes collected through sensors. For instance, if the system detects that an adolescent is on the move, it could ask where they are going. By developing a dynamic system that sends customized prompts based on the daily life and behaviors of adolescents, we can potentially bridge strong relatedness with the system.

As technology evolves, the acquisition of digital phenotypes becomes increasingly feasible, offering promising benefits for mental health monitoring and intervention. However, along with these opportunities come inherent challenges, particularly in data collection. Our study tackles these challenges by proposing effective strategies for collecting active digital phenotypes, especially among adolescents, and validating their feasibility through field simulations. These findings provide valuable insights for researchers and practitioners intending to collect digital phenotypes within adolescent populations.

### Limitations and Future Work

While our results show promise, there are some limitations to consider. First, the sustainability of the high compliance observed in our study remains uncertain in a real-world field setting. Moreover, this study was conducted for only 2 weeks, which is a short period to assess adherence comprehensively. Our reliance on a simulation tool indicates a potential gap when transitioning to a working prototype, necessitating validation with a fully developed system over an extended period. Future research should retain the factors that contributed to the high adherence observed in this study while exploring elements such as rewards, gamification, and collaborative goal setting to stimulate motivation in adolescents.

Second, the skewed participant demographic toward Korean female adolescents raises concerns about the generalizability of our findings. Considering that adolescents’ behaviors and emotions are influenced by culture, follow-up studies targeting groups with different demographic characteristics are necessary to ensure the extensibility of the results. In addition, none of the participants had received clinical diagnoses for mood disorders, which could affect the system’s applicability for monitoring and tracking mood disorders as opposed to daily emotional issues. To robustly validate the effectiveness of the system, it is necessary to support the results with studies involving clinically diagnosed individuals.

Furthermore, this study focused only on depression, anxiety, and stress among the many factors that constitute emotional issues in adolescents. Given the variety of mood disorders, this approach may be superficial in achieving a comprehensive understanding. Therefore, future research should address a broader range of emotional difficulties that adolescents face and include relevant symptoms in the EMA items. Moreover, integrating advanced technologies could enhance the collection of psychological data that traditional surveys might not capture. For example, allowing individuals to describe their emotional issues in written form while answering the EMA could facilitate more detailed text analysis.

Although we acknowledge the challenge of passive phenotype data collection, this issue was not sufficiently addressed in our study. It is essential to recognize that both active and passive data complement each other, and future research should aim to explore and incorporate passive data collection methods to enhance the comprehensiveness of our findings. Lastly, while some adolescents found value in self-recording for managing behavior and mood issues, our system primarily emphasized data collection rather than intervention. Future efforts could expand the system’s interventional potential by extending self-recording capabilities to include strategies aimed at promoting active coping behaviors and enhancing emotional well-being management within the system’s design goals.

### Conclusions

In conclusion, our study introduces promising concepts for designing an active digital phenotype collection system tailored to adolescents. We emphasize the importance of integrating seamless and user-friendly recording methods that align with adolescents’ natural behavior patterns. This approach not only simplifies data collection but also fosters a stronger sense of connection with the system by reflecting the daily routines of adolescents. The positive feedback and high compliance rates observed in our proof-of-concept study provide compelling evidence of the effectiveness of these concepts in capturing active phenotypes from adolescent participants.

## Abbreviations

EMA: ecological momentary assessment

## Data Availability

The datasets generated during and/or analyzed during this study are available from the corresponding author on reasonable request.
